# *Smallanthus sonchifolius* (Yacon) Flour Improves Visceral Adiposity and Metabolic Parameters in High-Fat-Diet-Fed Rats

**DOI:** 10.1155/2018/5341384

**Published:** 2018-10-28

**Authors:** Stella Maris Honoré, Maria Virginia Grande, Jorge Gomez Rojas, Sara Serafina Sánchez

**Affiliations:** Instituto Superior de Investigaciones Biológicas (INSIBIO), Consejo Nacional de Investigaciones Científicas y Técnicas-Universidad Nacional de Tucumán (CONICET-UNT), Chacabuco 461, T4000ILI San Miguel de Tucumán, Argentina

## Abstract

*Smallanthus sonchifolius* (yacon), a native plant of South America, was observed to improve lipid profile in rodents and humans. This study aimed to investigate the antiobesity properties of yacon roots in a high-fat-diet (HFD) model and the underlying mechanisms. A total of 30 Wistar male rats were divided into five groups (*n*=6): the standard chow diet (SD) group was fed a SD; the HFD group was fed a HFD; and the HFD Y340 and HFD Y680 groups were fed a HFD plus yacon flour (340 and 680 mg FOS/kg b. w./day, respectively). HFD Y340 and HFD Y680 rats exhibited marked attenuation of weight gain, a decrease in visceral fat pad weight, a restoration of the serum lipid profile and atherogenic index in a dose-dependent manner, being the higher dose more effective (*p* < 0.05). In addition, we found that HFD Y680 rats showed lower glucose and insulin levels, improved glucose tolerance, and insulin sensitivity (*p* < 0.5). A downregulation of several adipocyte specific-transcription factors, including peroxisome proliferator-activated receptor gamma2 (PPAR-*γ*2), CCAAT/enhancer binding protein a (C/EBP-a) and activating protein (aP2) mRNA levels, was determined in the visceral adipose tissue of HFD Y680 rats (*p* < 0.05). An improvement of adipokine profile in HFD Y680 rats and decreased serum proinflammatory cytokine levels (*p* < 0.05) were determined by ELISA. Decreased macrophage infiltration and F4/80 and MCP-1 expression in the visceral adipose tissue of HFD Y680 rats (*p* < 0.5), together with a higher pAkt/Akt expression (*p* < 0.05) were also observed by immunofluorescence and immunoblotting. A significant increase in glucagon (Gcg) and PYY mRNA levels in distal ileum of HFD Y680 rats (*p* < 0.05) were also detected. In the second approach, we determined that yacon supplementation potentiates the effects of the HFD reversion to a standard diet. In conclusion, yacon showed antiobesity properties by inhibiting adipogenesis and improving the visceral adipose tissue function.

## 1. Introduction

Overweight and obesity have become a global health problem owing to their strong association with a high incidence of various chronic diseases, such as type-2 diabetes, hypertension, coronary heart disease, and other noncommunicable diseases [1]. Obesity results from an energy imbalance between calorie intake and energy expenditure. The extra energy is stored as triglyceride in adipose tissue through an adipogenic process and accumulated in ectopic sites like muscle and liver, leading to a metabolic dysfunction [2, 3].

Adipogenesis is a process of cell differentiation by which precursor mesenchymal cells give rise to mature adipose cells to fulfill a key metabolic and endocrine role. Different hormones, nutrients, and transcription factors have been shown to regulate lipid accumulation during adipocyte differentiation [4]. Furthermore, the regulation of adipogenic transcriptional factors of mRNA levels, such as peroxisome proliferator-activator receptor-*γ* (PPAR-*γ*), CCAAT/enhancer binding protein-*α* (C/EBP-*α*), and related genes (adipocyte fatty acid-binding protein: AP2; fatty acid synthase: FAS), leads to changes in the activity of key proteins involved in lipids and fatty acids metabolism, inflammation, and cell-cycle regulation [5].

During obesity, adipose tissue expansion is marked not only by an increase in white adipose tissue mass but also by morphological changes which affects adipocyte function [6, 7]. Increasing evidence indicates that the excessive fat depots, particularly visceral adiposity, are linked to a chronic low-grade inflammatory state [1, 4, 5]. Moreover, through the release of free fatty acids (FFAs) and various adipokines such as leptin, adiponectin, resistin, PAI-1, Il-1b, and TNF-*α*, the hypertrophic adipocytes play a role in the progression of insulin resistance [2, 8].

It is known that obesity is not always spontaneously reversible, so its treatment requires a multidisciplinary approach [9]. The combination of diet and physical activity is only effective during time in which it is practiced and the available drugs are only moderately effective and have significant adverse effects [10, 11]. Dietary supplements have been proposed to promote weight loss or appetite suppression in the management of obesity. In this sense, natural products provide a rich source for safe and effective therapeutic compounds with multiple mechanisms of action [12, 13].

Yacon (*Smallanthus sonchifolius* (Poepp and Endl.) H. Robinson) belongs to a member of Asteraceae family, which ranges through the Andean region in South America [14, 15]. Yacon roots have special features which include high water content and large amount of soluble dietary fibers, with low energy density [14]. Given their high content in fructooligosaccharides (FOSs) [16, 17] and phenolic compounds, such as chlorogenic and caffeic acids [18], yacon roots have been considered as a beneficial functional food with prebiotic properties [19–22]. FOSs are fructose oligosaccharide joined by *β* (2 → 1) or *β* (2 → 6) linkages, able to resist the hydrolysis of enzymes in the upper gastrointestinal tract. Experimental studies have demonstrated that the addition of oligofructose to the diet improves the growth of *Bifidus* and *Lactobacillus* in the colon enhancing mineral absorption and gastrointestinal metabolism in both humans and animals [16, 19]. Dietary polyphenols also modulated the growth of beneficial microbial populations, influencing the intestinal mucosa integrity and energy harvest, through endocrine and systemic metabolic signaling [23, 24].

Previous studies demonstrated that dietary yacon supplementation reduces postprandial serum triglycerides in normal rats, without toxicity or adverse nutritional effects [17]. Also, it was shown that yacon improves beta cell function and modulates the plasma insulin concentration in diabetic rats [25, 26]. Additionally, yacon roots present strong antioxidant activity and anti-inflammatory effects preventing the risks associated with metabolic diseases [27–29]. Recently, long-term consumption of yacon syrup has been shown to improve insulin resistance and reduce body weight in premenopausal women [30]. These findings raise the interesting possibility that adipose tissue could be a target organ of the yacon roots in the management of obesity. However, no data are currently available on the ability of yacon roots to affect adipose tissue.

A number of studies have shown that the diet-induced obese animal models mimic human obesity more than other models such as genetic knockout mutants [31]. For instance, rodents chronically exposed to a high-fat diet develop dyslipidemia, white adipose tissue expansion, insulin resistance, and altered metabolic regulatory hormones [32, 33] constituting a useful tool to evaluate the potential mechanisms underlying the effects of yacon on obesity.

The current study was designed to assess efficacy of *Smallanthus sonchifolius* roots in suppressing visceral fat accumulation, ameliorate obesity-related phenotypic and biochemical markers, and provide a molecular mechanism for how yacon dietary supplementation can improve obesity in a HFD-fed-rat model. Even more is investigated if the effects of yacon are modified by the type of diet consumed.

## 2. Materials and Methods

### 2.1. Plant Material and Root Flour Preparation

The *Smallanthus sonchifolius* (yacon) (Clone LIEY97-1) roots, are cultivated locally at 550 m above the sea level, in the province of Tucumán, 27S, NW Argentina. Voucher specimens were deposited in the herbarium of “Instituto Miguel Lillo,” San Miguel de Tucumán, Tucumán, Argentina (No. 600982LIL). The roots were carefully washed, peeled, sliced, and dried at 60°C in a forced air circulation oven to reduce water content. The dried material was then pulverized to obtain yacon roots flour. The powder was stored at 4°C until use.

### 2.2. Carbohydrate Composition and Phenolic Content of Yacon Flour

Total sugar content and carbohydrate composition of yacon flour were estimated in samples extracted with 80% ethanol at 80°C [34]. Total sugar content of yacon flour was estimated by the phenol-sulphuric acid method [35]. The extract was dried, dissolved in water, and desalted with a mixed exchange resin (Amberlite MB3, Sigma). The purified water extract was injected into an HPLC system equipped with an IR detector (Gilson 132 IR) using a RSO-oligosaccharide Ag^++^ column (Rezex) and water at 70°C as the mobile phase. Oligosaccharides peaks were identified using sucrose, glucose, fructose, and fructofuranosylnystose as external standards [25]. In the experimental designs, daily intake levels of yacon root flour were calculated with respect to the amount of FOS using doses equivalent to 340 mg FOS/Kg b. w./day [25] and 680 mg FOS/Kg b. w./day corresponding to 0.79 and 1.57 g of yacon flour/kg body weight/day, respectively.

Total polyphenol content was determined by the Folin–Ciocalteu method [36] and expressed as milligram gallic acid (GAE) equivalents per gram of flour. Results of the analysis for the yacon flour are shown in Table 1.

### 2.3. Animals and Diets

Male Wistar rats weighing 200–250 g were obtained from the colony bred at the INSIBIO (CONICET-UNT), Tucumán, Argentina. Rats were kept in a breeding room with controlled environment (temperature: 23 ± 1°C, relative humidity: 60 ± 5%, and 12 h light-dark cycle). All the experimental procedures were in strict accordance with the Guide for the Care and Use of Laboratory Animals (Institute of Laboratory Animal Resources, Commission on Life Sciences, National Research Council, National Academy Press, Washington, DC) and the local Animal Care Committee from the Universidad Nacional de Tucumán (Prot. No. 004/2017).

**Figure 1:**
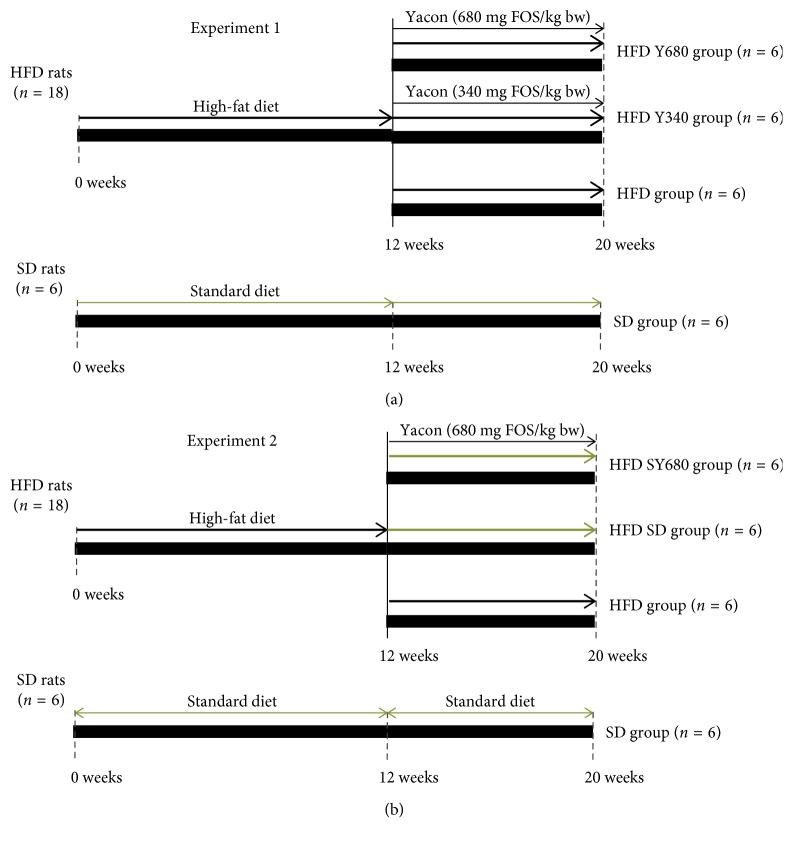
Experimental design. (a) Experiment 1: effects of the yacon flour on HFD-induced metabolic disease. (b) Experiment 2: effects of yacon supplementation and the reversion to a SD diet on HFD-induced metabolic disease. SD: standard diet-fed rats; HFD: high-fat-diet-fed rats; HFD SD: high-fat-diet-fed rats reversed to a SD diet; HFD SY680: high-fat-diet-fed rats reversed to a SD diet and supplemented with yacon flour (680 mg FOS/kg b.z.).

The experimental animals were randomly divided into two groups: the standard diet group (SD, *n*=12) and the high-fat-diet group (HFD, *n*=36). The SD group was fed *ad libitum*, with a standard chow diet containing 12.08 kJ/g calories: 69.5% from carbohydrates, 5.6% from fat, and 24.9% from protein (Association de Cooperativas Argentinas-S.E.N.A.S.A. No. 04-288/A). The HFD group received a standard-based diet enriched with eatable lard and carbohydrates (modified from [33]) to induce obesity and hyperlipidemia. The total calories in fat diet were 17.40 kJ/g: 35.0% from carbohydrates, 41.0% from fat, and 24.0% from protein. Both animal groups were maintained on each diet for 12 weeks. At this time, animals fed HFD reached obesity status (Figure 1).

### 2.4. Experimental Design

#### 2.4.1. Experiment 1

After 12 weeks on HFD or standard chow, the animals were randomly divided into the following groups according to the treatment with or without the addition of yacon flour as a dietary supplement for 8 weeks (Figure 1).

- HFD Y340 group (*n*=6), rats fed a high-fat-diet plus a tablet of yacon flour equivalent to 340 mg FOS/kg b. w.
- HFD Y680 group (*n*=6), rats fed a high-fat-diet plus a tablet of yacon flour equivalent to 680 mg FOS/kg b. w.
- HFD group (*n*=6), rats fed a high-fat-diet.
- SD group (*n*=6), rats fed standard diet.

#### 2.4.2. Experiment 2

In a second design, after 12 weeks rats which fed standard diet continued with the same chow, while animals under HFD were changed to a standard diet with or without yacon, according to the following scheme (Figure 1).

- HFD SD group (*n*=6), rats which change high-fat-diet feeding by standard diet
- HFD SY group (*n*=6) rats which change high-fat-diet feeding by fed standard diet plus a tablet of yacon flour containing 680 mg FOS/kg b. w.
- HFD group (*n*=6), rats fed a high-fat-diet.
- SD group (*n*=6), rats fed standard diet.

### 2.5. Morphometric and Nutritional Determinations

Animals were weighed (g) weekly throughout the experimental period. Food intakes were recorded daily as follows: food intake = initial food weight (g) – leftover food weight (g) – spilled food weight (g); energy intake = food intake (g) x total energy of the chow diet (kJ/g). The spilled food was weighed and dried after feces had been picked out. The body length (nose–anus length), thoracic circumference (immediately behind the foreleg), and abdominal circumference (immediately anterior to the forefoot) were determined in all rats at as was described previously [37]. The body weight and body length were used to determine the following anthropometrical parameters: Body mass index (BMI) = body weight (g)/length^2^ (cm^2^); Lee index = cube root of body weight (g)/nose-to-anus length (cm).

Nutritional parameters were calculated based on food and caloric intake: energy intake (kJ/day) = mean food consumption x dietary metabolizable energy; FER (food efficiency ratio) = (body weight gain/food intake) ×100.

### 2.6. Tissue Sampling

At the end of the experimental period, rats were fasted overnight and deeply anaesthetized with 1 :1 xylazin-ketamine. Blood samples were collected by cardiac puncture into EDTA-Na 4.1%-containing tubes, and plasma was separated by centrifugation at 3000 g for 10 min [25]. Blood samples were collected into plane glass tubes. After clotting, samples were centrifuged and serum was used for biochemical determinations. Liver, muscle (soleus), spleen, pancreas, and visceral (mesenteric), perirenal, and epididymal fat pads were removed and rinsed thoroughly with ice-cold saline, blotted, weighed, and fixed in 4% formaldehyde saline for histological analysis. The remaining tissues were frozen immediately and stored at −80°C until analyzed.

### 2.7. Biochemical Determinations

Triglycerides and total cholesterol concentrations were determined by enzymatic colorimetric methods using available commercial kits (Wiener lab Group, Argentina). The high-density lipoprotein cholesterol (HDLc) was determined after precipitation of very low-density lipoprotein (VLDL) and low-density lipoprotein (LDLc) with polyanions (dextran sulphate and magnesium chloride). LDL-cholesterol concentration was determined by a two-step homogeneous assay without precipitation. Free fatty acids were determined using available commercial kits (ab65341, Abcam, USA).

Circulating tumor necrosis factor-*α* (TNF-*α*) (RAB0479), interleukin-1*β* (IL-1*β*) (RAB0277), insulin (RAB0904) (all from SIGMA Aldrich, St. Louis, MO, USA), leptin (ab100773), and adiponectin (ab108784) (Abcam, USA) were performed using commercially available kits according to the manufacturer′s instructions.

Blood glucose concentrations were measured using a glucose meter (Roche Diagnostics GmbH, Mannheim, Germany). The HOMA-IR (Homeostasis model assessment of insulin resistance) index was calculated as [fasting glucose (mg/dl) × fasting insulin (ng/ml)/405] to assess insulin resistance [38].

#### 2.7.1. Oral Glucose Tolerance Test (OGTT)

Rats were administered orally with 50% D-glucose (2 g/kg body weight) after a 12 h fast. Blood glucose concentration was measured with a Glucometer (Accu-Check; Roche Diagnostics, GmbH, Mannheim, Germany) with blood from tail-tip bleedings at 0, 15, 30, 60, and 120 min. Area under the curve (AUC) was calculated as changes from 0 to 120 min and expressed in (mg/dl/min).

#### 2.7.2. Insulin Tolerance Test (ITT)

Rats were injected intraperitoneally with 0.75 IU/kg body weight porcine Insulin (Betasint, BETA laboratory) after a 4-hour fast. Blood glucose concentration was measured with a Glucometer (Accu-Check; Roche Diagnostics, GmbH, Mannheim, Germany) with blood from tail-tip bleedings at 0, 15, 30, and 60 min. The areas under the curve (AUC 0–60 min) were calculated.

#### 2.7.3. Oral Fat-Loading Test

To assess postprandial increase in triglyceride, oral fat-load tests were performed at the end of 8 week of yacon diet-supplementation. Rats from the HFD Y680, HFD, and SD groups were fasted for 12 h. The HFD Y680 group received a single yacon-tablet containing 680 mg FOS/kg body weight. Thirty minutes later with 2.5 ml/kg b. w. of corn oil was given to all groups of animals. Tail blood samples were taken at 0 h and 1, 2, 3, and 4 hours after oral chow administration for the triglycerides determination. AUCs (0–4 h) were calculated in both experiments.

### 2.8. Histology and Immunohistochemistry

Samples from fixed visceral fat were dehydrated, embedded in paraffin, and cut into 4 *μ*m-thick sections at 50 *μ*m intervals. The sections were stained with hematoxylin and eosin (H&E), mounted on glass slides, and examined by optical microscopy. Images were analyzed using ImageJ software for quantification (National Institutes of Health, Bethesda, MD).

After, blocked with 10% (w/v) normal goat serum for 1 h, sections were subjected to immunohistochemical staining overnight at 4°C with a 1 : 100 dilution of a anti MCP-1 polyclonal antibody (Santa Cruz Biotechnology, USA) following by Alexa Fluor 594 antibody (Invitrogen) (1 h, at room temperature). The sections were mounted in aqueous mounting medium with antifading agents (Biomeda, Foster, CA). The specimens were analyzed using an Olympus BX80 fluorescence microscope (Olympus Optical Ltd., Tokyo) and the ImageJ software. At least 15 nonadjacent microscope fields were analyzed in each tissue.

### 2.9. RNA Extraction and PCR and qPCR Amplification

Total RNA was isolated from visceral adipose and intestine tissue using the RNeasy Lipid Tissue Mini Kit (Qiagen, Basel, Switzerland) and Trizol reagent (Invitrogen), respectively, according to the manufacturer′s instruction. 0.5 *μ*g RNA was reverse transcribed (RT) into first-strand cDNA using M-MLV Reverse Transcriptase (Promega, USA) and oligo (dT) primers (Invitrogen, USA). After the RT procedure, the resulting cDNAs were used for PCR and qPCR. Gene expression was evaluated using a Mastercycler personal instrument (Eppendorf, Germany) in optimized conditions and quantified using the QuantiFast SYBR Green Kit (Qiagen, Hilden, Germany) on a Lightcycler 2.0 instrument (Roche Diagnostics, Mannheim, Germany) with the following cycle conditions: 95°C for 10 seconds followed by 30 cycles of 95°C for 5 seconds and 57°C for 30 seconds. The primers used are PPAR*γ*2 (AB019561.1) forward primer 5′-CCCTGGCAAAGCATTTGTAT-3′ and reverse primer 5′-ACTGGCACCCTTGAAAAATG-3′; C/EBP-a (NM001287577) forward primer 5′-GGAGGGACTTAGGGAGTTGG-3′ and reverse primer 5′-GGAAACCTGGCCTGTTGTAA-3′; aP2 (U75581) forward primer 5′-GGGACCTGGAAACTCGTCTC-3′ and reverse primer 5′-CTGACCGGATGACGACCAAG-3′; Gcg (NM012707.2) forward primer 50-CATTCACAGGGCACATTCAC-30 and reverse primer 50-TGACGTTTGGCAATGTTGTT-30; PYY (AB238226.1) forward primer 50-GTGGACCAGTGGTGAAGACC-30 and reverse primer 50-GGGACATGAACACACACAGC-30; *β*-actin (NM007393) forward primer 5′-CCGGCTTCGCGGGCGACG-3′ and reverse primer 5′-TCCCGGCCAGCCAGGTCC-3′. The results were normalized to *β*-actin mRNA levels.

### 2.10. Western Blotting

Visceral adipose tissues were homogenized in the whole cell lysis buffer containing 1% NP-40, 0.1% SDS, 0.5% sodium deoxycholate (SIGMA Aldrich, USA), 0.1 mM EDTA, 2 mM PMSF, a Complete Protease Inhibitor Cocktail (Roche, Germany), and supernatants were collected. An amount of 25–50 mg of protein was loaded on to 7.5% SDS polyacrylamide gels and transferred to 0.22/0.45 mm a nitrocellulose membrane (Hybond-C super; Amersham, Buckinghamshire, UK). The membrane was blocked with 5% (w/v) fat-free milk dissolved in phosphate buffered saline containing 0.05% (v/v) Tween-20 (TBST). The membrane was incubated at 4°C overnight with rabbit polyclonal antibody against Akt (1 : 100 dilution; Cell Signaling Technology Inc. Danvers, USA), p-Akt (1 : 100 dilution; Cell Signaling Technology Inc. Danvers, USA), F4/80 (1 : 100 dilution; Santa Cruz Biotech, USA), or Actin (1 : 3000 dilution; SIGMA Aldrich, USA). The washed membrane was incubated with HRP-conjugated secondary antibodies at room temperature for 1 h, and then a biotin-extrAvidin-peroxidase system (SIGMA Aldrich, USA) was used to determine signals. Band intensities were quantified by ImageJ software.

### 2.11. Statistical Analysis

All results are presented as the mean ± standard deviation and were analyzed with Graph Pad Prism 6.01 (San Diego, CA, USA). To assess the significance of variation, groups were compared by one-way ANOVA followed by Bonferroni′s multiple comparisons test. A probability level of *p* < 0.05 was considered statistically significant.

## 3. Results

### 3.1. Yacon Improves Morphometric and Feeding Parameters in HFD-Fed Rats

**Figure 2:**
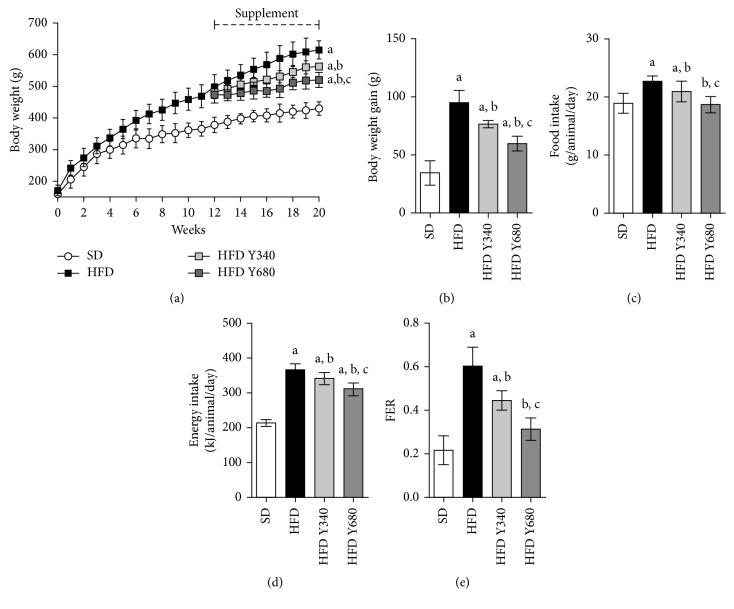
Effect of yacon flour on HFD-fed rats after  8 weeks of supplementation. (a) Time course of body weight in rats fed standard diet (SD) or high-fat diet (HFD) supplemented or not with yacon flour (340 or 680 mg FOS/kg body weight: HFD Y340 and HFD Y680, respectively). (b) Body weight gain. (c) Food intake. (d) Energy intake. (e) Feed efficiency ratio (FER). Data are mean ± standard deviation (*n*=6/group). ^a^ *p* < 0.05 vs. SD group, ^b^ *p* < 0.05 vs. HFD group, ^c^ *p* < 0.05 vs. HFD Y340 group.

Rats fed a high-fat diet greatly increased the body weight compared to the SD group (*p* < 0.05). The average growth rate for rats on the HFD was 19.7 g/week while rats on a SD-chow were merely 10.2 g/week. At  12 weeks, the body weight of HFD rats resulted around 20% higher compared with SD-fed rats (*p* < 0.05) (Figure 2(a)). Yacon flour supplementation to the HFD rats for the following 8 weeks significantly reduced the final body weight in a dose-dependent manner (*p* < 0.05) (Figures 2(a) and 2(b)). An improvement in the morphometric parameters was also observed in supplemented rats (Supplemental data, Table S1). Yacon significantly reduced the food intake leading to a lower energy intake (*p* < 0.05) (Figures 2(c) and 2(d)). Food efficiency ratio (FER) was also reduced in both tested doses compared with the HFD group (*p* < 0.05) (Figure 2(e)).

### 3.2. Yacon Improves Lipid Serum Profiles in HFD-Fed Rats

Fasting triglycerides (TG), low-density lipoprotein (LDLc), and free fatty acids levels were increased in HFD-fed rats compared with the SD group, whereas high-density lipoproteins (HDLc) were reduced (*p* < 0.05). Yacon significantly reduced triglycerides and free fatty acids concentrations in both doses tested with a higher effect at 680 mg FOS/kg b. w. (*p* < 0.05), evidencing a dose-dependent effect on these parameters. No effect on total cholesterol levels was observed (*p* > 0.05). Interestingly, yacon flour significantly decreased LDLc and increased HDLc values at the dose of 680 mg FOS/kg b. w, improving the TG/HDLc index (*p* < 0.05) (Table 2).

### 3.3. Yacon Improves Body Composition of HFD-Fed Rats

Table 3 shows the effects of yacon flour consumption on relative organ weights. HFD-fed rats exhibited an increase in hepatic mass and a decrease in the cecum and muscle (*p* < 0.05). Administration of yacon supplement reduced the liver mass in HFD rats in both tested doses (*p* < 0.05). Only the dose of 680 mg FOS/kg b. w. was able to increase significantly the cecum weight. There was no alteration in the soleus weight after yacon supplementation (*p* > 0.05). No significant changes were observed for the spleen and pancreas in all the studied groups.

Yacon supplementation significantly reduced the total fat weight in HFD-fed rats at a dose of 680 mg FOS/kg b. w. (*p* < 0.05). However, only a tendency to decrease was shown at 340 mg FOS/kg b. w. dose. Interestingly, differences in fat deposition in terms of epididymal, perirenal, and mesenteric pads, were observed after yacon supplementation. Yacon considerably reduced visceral pad weight in both doses tested, with a maximum effect at 680 mg FOS/kg b. w. (*p* < 0.05) (Table 3). However, epididymal and retroperitoneal pads deposition decreased only at a higher dose of 680 mg FOS/kg b. w. (*p* < 0.05) (Table 3). These data led us to believe that visceral fat may be a target of yacon effects.

### 3.4. Yacon Modulates Adipocyte Size and Gene Expression in Adipose Tissue

**Figure 3:**
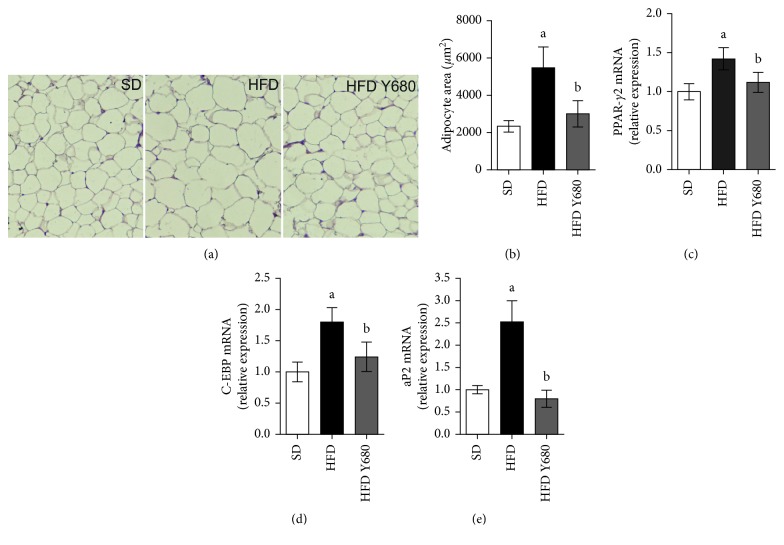
Effects of yacon flour on the adipose tissue of HFD-fed rats and adipogenesis-related genes. (a) Representative photomicrographs of visceral adipose tissue sections stained with H&E (original magnification 126X). (b) Adipocytes size in visceral adipose tissue. qPCR analysis of visceral fat from rats fed standard diet (SD) or high-fat diet (HFD) supplemented or not with yacon flour (680 mg FOS/kg body weight: HFD Y680) after  8 weeks of treatment: PPAR-*γ*2: peroxisome proliferator-activated receptor gamma2 (c); C/EBP-a: CCAAT/enhancer-binding protein (d); aP2: activating protein 2 (e). Data were normalized to Actin mRNA and expressed as fold change over the SD rats. Values are presented as mean ± standard deviation of triplicate qPCR analysis (*n*=6/group). ^a^ *p* < 0.05 vs. SD, ^b^ *p* < 0.05 vs. HFD.

At the cellular level, obesity is characterized by an increase in the number (hyperplasia) and size of individual adipocytes that have differentiated from preadipocyte in fat depots [5]. Histological analysis from visceral adipose tissue sections stained with H&E revealed that high-fat feeding increased fat deposits and adipocytes size in the HFD animals. Interestingly, yacon supplement (680 mg FOS/kg b. w.) resulted in reduced fat deposits and smaller adipocytes in visceral pad, with similar size to those observed in SD-fed rat (Figures 3(a) and 3(b)).

To analyze if yacon could modulate the expression of genes involved in adipogenesis in visceral fat, the amount of PPAR-*γ*2, a major transcription regulator of adipogenic process, was measured in supplemented HFD-fed rats. The expressions of PPAR-*γ*2, as well as their downstream targets, C/EBPa and aP2, were significantly decreased in HFD Y680-fed rats (*p* < 0.05) (Figures 3(c)–3(e)). These data imply a role of yacon on avoiding visceral adipose tissue expansion induced by diet.

### 3.5. Yacon Reduces Inflammation in Visceral Adipose Tissue of HFD-Fed Rats

**Figure 4:**
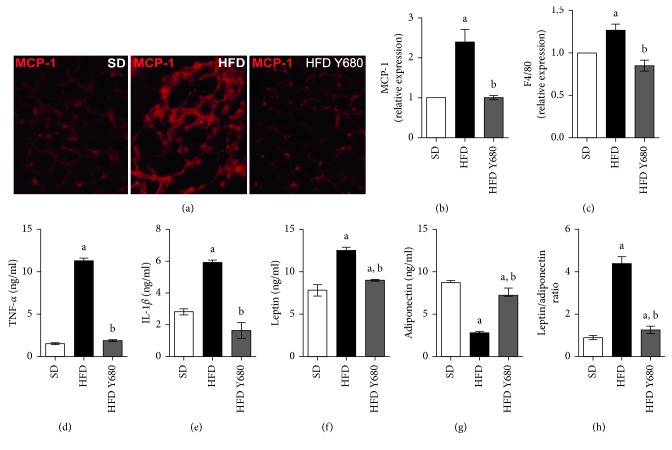
Effects of yacon flour on inflammatory markers in HFD-fed rats. (a) Representative sections of visceral fat stained with MCP-1 from rats fed standard diet (SD) or high-fat diet (HFD) supplemented or not with yacon flour (680 mg FOS/kg body weight: HFD Y680) (original magnification 126X). (b) Staining score, assigned to each section according to the MCP-1 levels. (c) F4/80 protein expression of in visceral fat by western blotting. (d, e) Serum TNF-*α* and IL-1*β* concentrations determined by ELISA. (f, g) Plasma leptin and adiponectin concentrations. (h) Leptin/adiponectin ratio. Data are mean ± standard deviation (*n*=6/group). ^a^ *p* < 0.05 vs. SD, ^b^ *p* < 0.05 vs. HFD.

It is known that excessive adipose tissue expansion leads to adipose macrophage infiltration and metabolic dysfunction contributing to low-grade local and systemic inflammation [2]. In this way, we analyzed whether yacon could modulate inflammatory markers in visceral fat and serum. MCP-1 expression levels and the macrophage biomarker F4/80 were significantly elevated in the visceral adipose tissue of HFD-fed rats compared with SD rats (*p* < 0.05). Yacon supplementation reduced the expression of the mentioned markers in HFD-fed animals (Figures 4(a)–4(c)). In accordance with this fact, yacon also reduced the circulating level of proinflammatory cytokines TNF-*α* and IL-1*β* levels in HFD-fed animals (Figures 4(d) and 4(e)).

Additionally, adiponectin and leptin have been implicated in adipose tissue dysfunction [6]. Therefore, the effect of yacon on modulating these adipokine levels in HFD rats was evaluated. High leptin and low adiponectin levels were observed in HFD-fed (*p* < 0.05). Yacon supplementation significantly decreased the leptin and increased the adiponectin concentrations in HFD-fed rats, restoring the leptin/adiponectin ratio (*p* < 0.05) (Figures 4(f)–4(h)).

### 3.6. Yacon Improves Metabolic Parameters in HFD-Fed Rats

**Figure 5:**
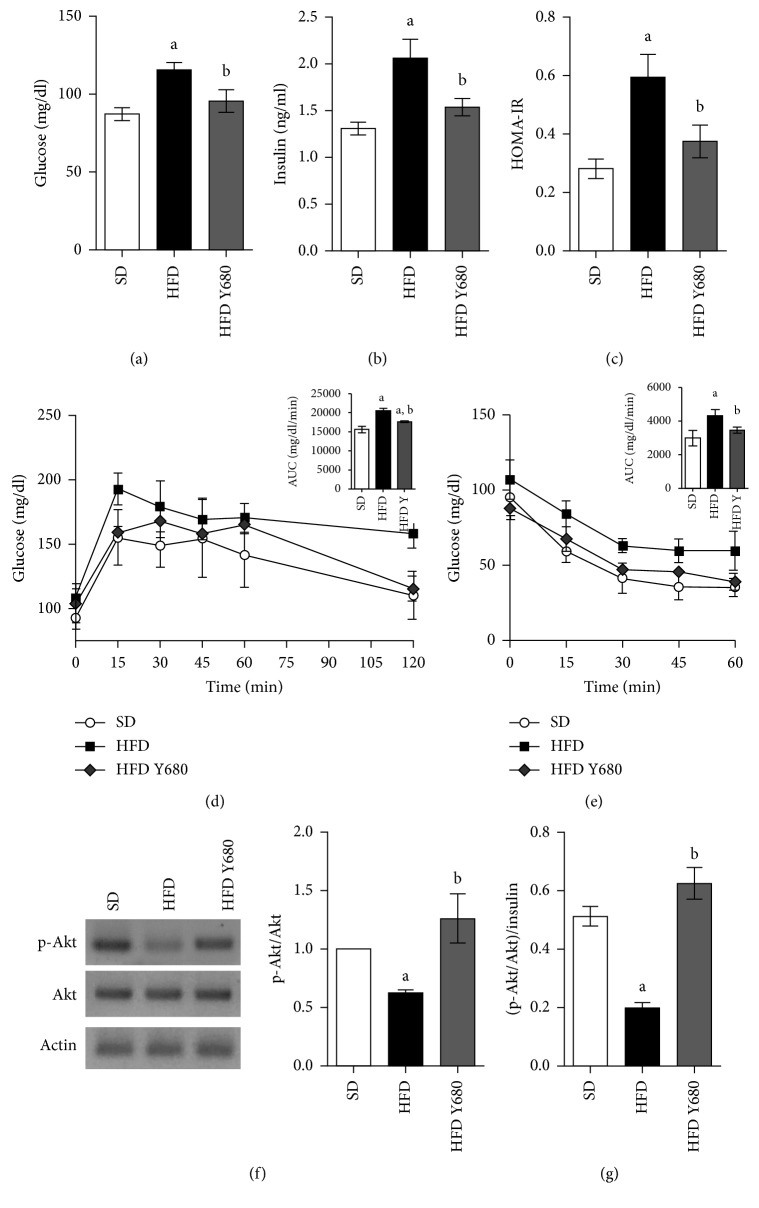
Effects of yacon flour on HFD-induced metabolic disease. (a) Fasting glucose concentrations at the end of experiment. (b) Time course of glycemia in response to oral glucose overload (2 g/kg body weight) at  8 weeks (insert: area under the curve of blood glucose following glucose overload). (c) Time course of glycemia following a single intraperitoneal (i.p.) injection of insulin (0.75 U/kg b. w.) after 8  weeks of yacon supplementation (insert: area under the curve of blood glucose following insulin injection). (d) Fasting plasma insulin concentrations at the end of experiment. (e) Homeostasis model assessment of insulin resistance (HOMA-IR) index. (f) Akt and p-Akt protein expressions in visceral fat by western blotting. The mean value of SD-fed rats was set at 1. (g) p-Akt/Insulin ratio. Data are expressed as the mean ± standard deviation (*n*=6/group). ^a^ *p* < 0.05 vs. SD, ^b^ *p* < 0.05 vs. HFD. SD: standard diet-fed rats; HFD: high-fat-diet-fed rats; HFD SD: high-fat-diet-fed rats reversed to a SD chow; HFD SY680: high-fat-diet-fed rats reversed to a SD chow and supplemented with yacon flour (680 mg FOS/kg b. w.).

Estimation of blood glucose, insulin, and HOMA-IR index in the experimental groups is depicted in Figure 5. The results showed that high-fat feeding led to alterations in glucose homeostasis inducing a significant increase in the fasting serum glucose and insulin levels, accompanied by the increased HOMA-IR index (*p* < 0.05). Yacon supplementation significantly decreased fasting glucose and insulin levels resulting in a lower HOMA-IR index compared with HFD rats (*p* < 0.05) (Figures 5(a)–5(c)).

In addition, during OGTT, SD rats increased the blood glucose level to a maximum, 15 min after the oral glucose loading, and then declined to the basal value (Figure 5(a)). Whereas, glucose peak in HFD rats was higher at 15 min and remained high over the 120 min. Interestingly, yacon supplementation elicited a significant decrease in the blood glucose level at 15 min and beyond when compared with HFD rats (Figure 5(d)). The AUC was significantly increased in the HFD group compared to the SD group (*p* < 0.05), but decreased in the yacon-treated group (*p* < 0.05). Moreover, the ITT showed that the blood glucose levels after insulin injection in the SD- and HFD Y680-fed groups were lower than those in HFD rats (*p* < 0.05), indicating that yacon improved glucose tolerance and insulin resistance in HFD rats (Figure 5(e)).

Changes in protein expression of Akt, the most important molecule of the insulin signaling in target organs, were measured using Western blot analysis (Figure 5(f)). Akt protein expression was similar in the visceral adipose tissue of all the analyzed groups (*p* > 0.05). However, while Akt phosphoryation in the visceral adipose tissue of HFD rats was lower compared to SD rats (*p* < 0.05), yacon supplementation increased the phosphoryation levels of this protein, in HFD rats leading to higher p-Akt/Akt and pAkt/Insulin ratios (*p* < 0.05) (Figures 5(g)).

### 3.7. Yacon Improves Glucagon and PYY mRNA Expression in HFD-Fed Rats

**Figure 6:**
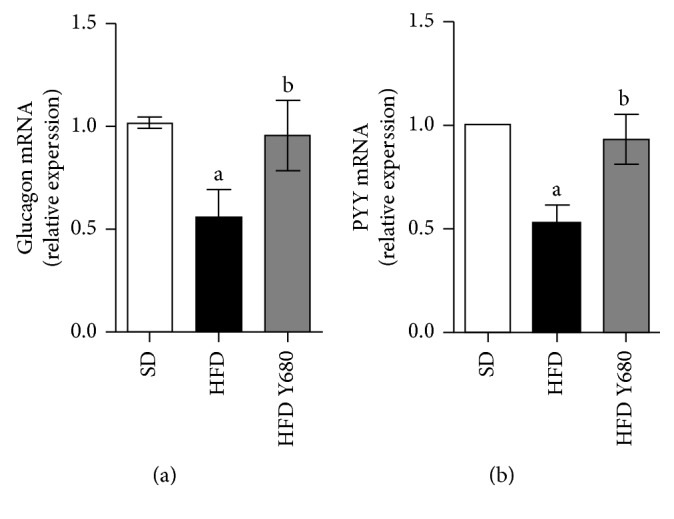
Effects of yacon flour on intestinal mRNA levels of proglucagon and PYY in HFD-fed rats. Proglucagon: Gcg (a) and polypeptide YY: Pyy (b) mRNA expression in intestinal samples of rats fed standard diet (SD) or high-fat diet (HFD) supplemented or not with yacon flour (680 mg FOS/kg body weight: HFD Y680) after  8 weeks of treatment. Data were normalized to Actin mRNA and expressed as fold change over the SD rats. Values are presented as mean ± standard deviation of triplicate PCR analysis (*n*=6/group). ^a^ *p* < 0.05 vs. SD, ^b^ *p* < 0.05 vs. HFD. SD: standard diet-fed rats; HFD: high-fat-diet-fed rats; HFD SD: high-fat-diet-fed rats reversed to a SD chow; HFD SY680: high-fat-diet-fed rats reversed to a SD chow and supplemented with yacon flour (680 mg FOS/kg b. w.).

It is well established that high-fat diet impairs gastrointestinal peptides secretion, implicated in appetite control and insulin release [31]. Then, we assessed whether yacon effects are related to changes in incretins expression. The mRNA levels of glucagon (Gcg), the precursor of glucagon-like peptide-1 (GLP-1) and the peptide tyrosine tyrosine (PYY) in distal ileum of the HFD-fed rats were significantly reduced compared to those of the SD-fed rats (*p* < 0.05). Dietary supplementation with yacon strongly increased Gcg and PYY expression of HFD-fed rats, recovering the mRNA levels (*p* < 0.05). The data concerning to the expression of intestinal peptides are shown in Figure 6.

### 3.8. Yacon Decreases Serum Triglyceride Levels after Oral Fat-Loading

Lipid intestinal uptake is a crucial step for obesity progression and hyperlipidemia. In order to assess whether the yacon modifies lipid absorption at the intestinal level, we perform an oral triglyceride loading test.  Figure S1 (Supplementary materials) shows the serum triglyceride values of the different experimental groups after oral oil loading. HFD rats showed a sharp increment of triglycerides at 30 min, reaching a maximum peak at 1h and then decreasing to triglyceride baseline value at 4 h. In contrast, the serum triglycerides curve of HFD Y680 rats did not increase considerably from the baseline, being similar to that from SD rats. These data indicate that yacon attenuates diet-induced obesity in rats by decreasing fat absorption.

### 3.9. Yacon Improves the Effects of the Reversion to Standard Diet

**Figure 7:**
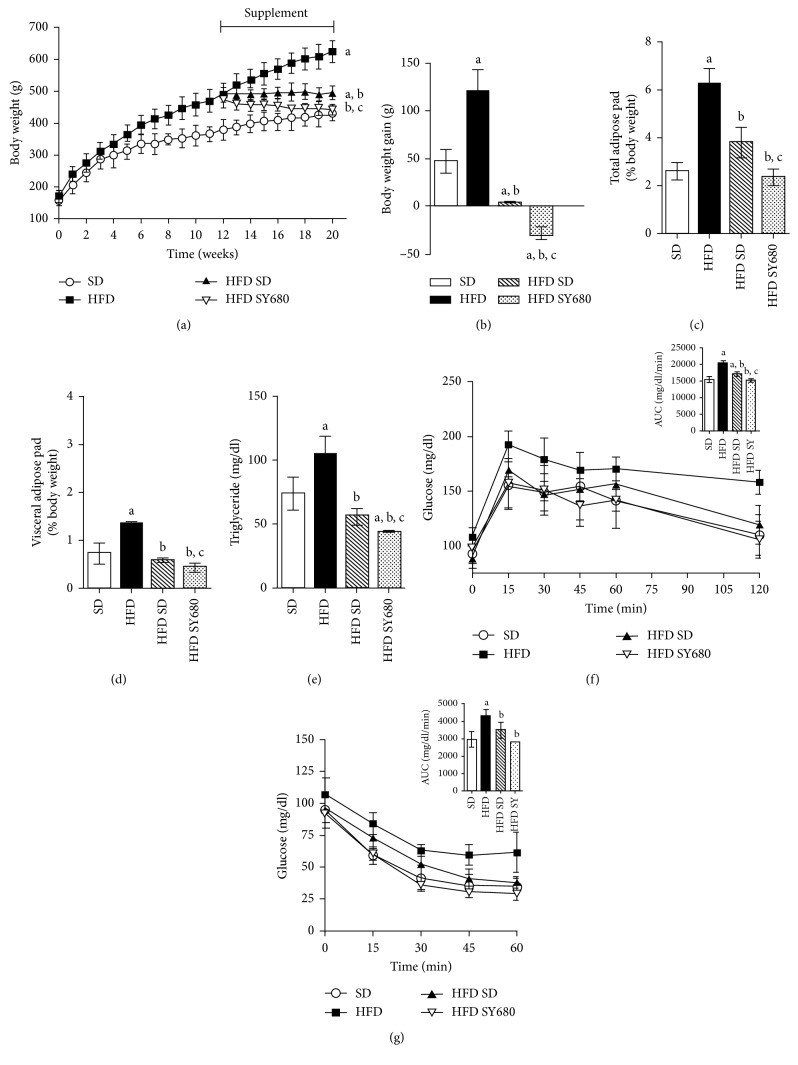
Effects of yacon supplement and the reversion to a SD chow on HFD-induced metabolic disease. (a) Body weight. (b) Body weight gain. (c) Total fat weight. (d) Visceral fat pad weight. (e) Fasting triglyceride concentrations. (f) Time course of glycemia in response to oral glucose overload (2 g/kg body weight) at 8 weeks (insert: area under the curve of blood glucose following glucose overload). (g) Time course of glycemia following a single intraperitoneal (i.p.) injection of insulin (0.75 U/kg b. w.) (insert: area under the curve of blood glucose following insulin injection). Data are mean ± standard deviation (*n*=6/group). ^a^ *p* < 0.05 vs. SD, ^b^ *p* < 0.05 vs. HFD, ^c^ *p* < 0.05 vs. HFD SD. SD: standard-diet-fed rats; HFD: high-fat-diet-fed rats; HFD SD: high-fat-diet-fed rats reversed to a SD chow; HFD SY680: high-fat-diet-fed rats reversed to a SD chow and supplemented with yacon flour (680 mg FOS/kg b.w.).

To identify whether the yacon supplementation potentiate the effects of the HFD reversion to a standard diet, we performed a second experimental design. We found that all of the parameters analyzed were improved after HFD rats turned to SD feeding for 8 weeks (Supplementary materials, Table S1). Interestingly, yacon consumption strongly reduced the final body weight and the body weight gain in the HFD SY680 group compared to HFD SD only (Figures 7(a) and 7(b)). Consistently, body and visceral adipose weight were significantly lower in HFD SY680-fed rats when compared with all the other groups (Figures 7(c) and 7(d)). Rats in the HFD SY680 group showed lower food intake than HFD SD rats, which resulted in a reduced energy intake and FER (Supplementary materials, Figure S2). Serum triglyceride levels of HFD SY680 rats were also significantly reduced when compared to HFD SD- or to SD-fed rats (Figure 7(e)).

In accordance with this finding, fasting glucose, glucose tolerance, and insulin resistance were also improved being the blood glucose concentrations similar to those of the SD rats during the glucose challenges (Figures 7(f) and 7(g)).

## 4. Discussion

Nutritional strategies represent alternatives to pharmaceutical approaches for reducing hyperglycemia and body weight [13]. Thus, the identification of novel foods that promote satiety or reduce energy density provides an interesting tool in managing obesity and its associated comorbidities [6, 13].

Here, we report an interesting effect of yacon root flour, a natural product rich in FOS and phenolic compounds, as a dietary supplement. The addition of yacon flour to a high-fat diet, at doses of 340 and 680 mg FOS/kg b. w./day, was effective in reducing body weight, in a dose-dependent manner. This effect was more pronounced when yacon was accompanied by a standard diet. Moreover, regional fat depots analysis showed primarily fat loss from the visceral pads, a known critical factor in reducing overall disease risk, including type-2 diabetes development [38]. Furthermore, *in vivo* effects of yacon roots on lipid metabolism included a significant reduction in serum triglycerides, VLDLc, and free fatty acids towards a healthy atherogenic index in HFD-fed rats, with a higher effect at 680 mg FOS/kg b. w.

One of the mechanisms by which the yacon root triggers weight loss seems to be a decrease in energy intake, given our observation of a lower food intake after yacon supplementation. Dietary fiber is known to attenuate food intake through different mechanisms that include the displacement of certain nutrients in the diet by the fiber; the inhibition of food absorption in the small intestine; and modification of satiety hormone response with appetite reduction [39]. It is well recognized that FOS could also be a fermentable substrate for the colon microbiota, being low polymerization FOS (DP2-10) the most used by *Bifidobacterium* spp. and *Lactobacillus* spp. [16]. Bifidobacteria have been shown to be beneficial to the health of the host and to be correlated with a lean state [40]. Bacterial fermentation of FOS in the cecum and proximal colon has been shown to produce short chain fatty acids such as acetate, propionate and butyrate. In addition, dietary polyphenols directly or indirectly impact on gut function, decreasing pathogenic bacterial growth and promoting the production of the microbial short chain fatty acids by beneficial microbial populations [23]. These carboxylic acids, especially butyrate, are also capable of stimulating the L-cells of the intestinal mucosa increasing the secretion of gut hormones that are involved in appetite regulation [41]. It has been reported that inulin-type fructans fermentation increase the production of the anorexigenic hormones glucagon-like peptide-1 (GLP-1) and peptide YY (PYY) in rodents affecting the food intake and energy expenditure [42, 43]. Consistently, in the present work, we reported an increased expression of Gcg (the GLP-1 precursor) and PYY genes in the distal ileum of yacon supplemented HFD rats. In concordance, increased GLP-1 levels in the colon and in serum were shown when yacon roots were administered to diabetic rats [25]. Thus, FOS and phenolic compounds present in yacon flour could be able to decrease body weight in HFD rats, through the central action of GLP-1 and PYY.

Chronic consumption of a high-fat diet has been shown to produce obesity characterized by a separate or simultaneous increase in adipocyte number and size [2]. Yacon roots had a significant effect on the reduction of visceral adipose tissue mass and adipocyte size. The expansion of adipose tissue begins when differentiated adipocytes rapidly respond to nutrient excess acting as lipid-synthesizing and lipid storing cells [5]. The differentiation of preadipocytes involves a highly regulated and coordinated cascade of transcription factors, where PPAR-*γ* and C/EBPa play a central role [4, 44]. PPAR-*γ*2 is the major regulator of adipogenesis and is also required for the maintenance of the adipocyte differentiated state [45]. PPAR-*γ*2, a splicing isoform of PPAR-*γ*, is selectively expressed in the adipose tissues. In powerful synergy with C/EBPa, PPAR-*γ*2 controls adipocyte differentiation and lipid metabolism by regulating transcription factors such as aP2 and lipogenic/lipolytic genes and thus enhances the efficiency of lipid utilization [46, 47]. aP2 is a carrier protein for fatty acids mainly expressed in adipocytes and macrophages and plays an important role in the development of insulin resistance and metaflammation [45]. In our study, the mRNA levels of the transcription factor PPAR-*γ*2 and its target genes C/EBPa and aP2 increased in the visceral adipose tissue of the HFD-fed rats. Similarly, previous reports indicated that PPAR-*γ*2 mediates high-fat-diet-induced adipocyte differentiation and adipocyte hypertrophy to generate large adipocytes and insulin resistance in obese patients [48] and in animal models [49]. Thus, decreased PPAR-*γ*, C/EBPa, and aP2 expression in visceral adipose tissue by yacon supplement may suppress adipogenesis, enhancing insulin sensitivity, and lowering blood lipid concentrations. Similarly, it has been reported that inulin-type fructans have an effect on differentiation and triglyceride accumulation in adipocytes through a decrease in PPAR*γ*-activated processes [50].

Increased adiposity during obesity greatly influences insulin sensitivity, glucose and lipid metabolism, and inflammation [3, 8, 45]. In the present study, rats long exposed to high-fat diet developed a hyperglycemic state associated with insulin resistance and/or glucose intolerance. Adiponectin is the most abundantly adipokine expressed in adipose tissue with pleiotropic insulin-sensitizing effects. It has been shown that it reduces hepatic glucose production and stimulates glucose uptake and fatty acid oxidation in skeletal muscle [51]. Leptin, another adipokine, regulates weight balance, glucose, and lipids metabolism [52]. While adiponectin levels reduce in response to excessive fat depots, plasma leptin concentrations are positively associated with the amount of body fat [53]. In our study, yacon supplement to obese animals modified adiponectin and leptin plasma concentrations increasing adiponectin/leptin ratio. Thus, the improved glucose tolerance and insulin action in obese rats after yacon supplementation may lead us to suggest that this beneficial effect could be mediated by adipokines modulation as was suggested previously [54]. Moreover, releasing GLP-1 from intestinal L-cells directly into the portal vein could also represent an additional mechanism related to yacon FOS-improved glucose homeostasis [55]. On the other hand, phenolic compounds are been involved in the regulation of postprandial glycemia and glucose tolerance also modulating gastrointestinal hormone secretion [56].

Obesity is now considered as a chronic inflammatory state linked to an abnormal cytokine production and the activation of inflammatory signaling pathways in adipose tissue [1, 3, 5]. The excess of nutrients induces the accumulation of macrophages, through the monocyte chemoattractant protein-1 (MCP-1), and is implicated in the development and maintenance of obesity-induced adipose tissue inflammation [57]. In the present study, we described an increased expression of MCP-1 and the macrophage marker F4/80 in the adipose tissue of obese animals which was significantly reduced by yacon supplement. This allows us to suggest that inhibition of MCP-1 by yacon supplement could lead to a low level of macrophage infiltration contributing to reduce adipose tissue inflammation improving insulin resistance and metabolic state. Moreover, our results also demonstrated that yacon treatment significantly decreased circulating levels of proinflammatory cytokines TNF-*α* and IL-1*β* in HDF-rats. This fact could be related to lower levels of TNF*α*, IL-1*β*, and MCP1 in the adipose tissues of supplemented HFD rats. Furthermore, it has been established that inflammatory cytokines TNF-*α* and IL-6 inhibit the expression of insulin signaling mediators in adipose tissue leading to a worsening of whole-body insulin resistance and glucose intolerance [1, 3]. Our results demonstrated that long-term yacon supplementation ameliorates abnormal insulin signaling cascades in the visceral adipose tissue of HFD rats by enhancing the phosphorylation level of the Akt protein, the pAkt/Akt ratio, and pAkt/Insulin ratio. In agreement with this, Satoh et al. [58] using a euglycemic-hyperinsulinemic clamp procedure demonstrated that yacon diet improved hepatic insulin sensitivity by increasing liver Akt activation in Zucker fa/fa rats.

High-fat diet is considered a direct cause in the development of dyslipidemia during obesity [31, 32]. The data obtained in the present study demonstrated that yacon flour administered to obese rats improved the lipids profile, reducing triglycerides and LDLc. It is interesting to note that the beneficial effects in lipids metabolism are more evident when yacon supplementation is accompanied by a standard diet. As previously reported by us, yacon roots exhibit a lipid lowering effect, mainly reducing serum triglycerides and VLDLc levels, in normal and diabetic rats [17, 25]. Moreover, in human studies, we demonstrate that yacon syrup lowers plasma lipid concentrations and decreases glycemia in patients with insulin resistance [30]. The triglyceride lowering effect could explain at least in part the inhibition of *de novo* fatty acid synthesis and downregulation of lipogenic enzymes as has been suggested for oligofructose [59]. In addition, it is well known that soluble dietary fibers delay fat absorption as we observed during the fat load test, contributing to the hypolipidemic effects of yacon.

On the other hand, the lack of significant changes in total cholesterol levels in our study may be explained by the short supplement-time and/or the concentration of FOS utilized in our study. Reductions in cholesterol have only been found with long-term feeding of oligofructose in relatively high doses [60]. Additionally, the presence chlorogenic and caffeic acid in yacon roots could also contribute to the hypolipidemic effect observed after yacon supplement [18]. It was established that caffeic acid, particularly chlorogenic acid, improves lipid metabolism in high-fat-diet-induced obese mice [61].

## 5. Conclusion

The data presented in this study suggest that yacon may act by different ways to suppress body weight gain, food intake, FER levels, weight and size of adipose tissue, and serum levels of triglyceride and improve adipokine profile in HFD-fed rats. These results can be associated with the decrease in adipogenesis related to mRNA expression of PPAR‐*γ*, C/EBPa, and aP2. The high content of nondigestible fermentable fibers present in yacon roots as FOS together with phenolic compounds could be largely responsible for the beneficial effects observed. This simple dietary intervention appears to be capable of improving obesity outcomes regardless of susceptibility to an obesogenic nutritional environment. Hence, yacon roots can be considered a potent and useful functional food.

## Figures and Tables

**Table 1:** Chemical characterization of yacon flour.

Component	Content
Total sugar (mg/g flour)	780.0 ± 5.24
FOS (mg/g flour)	432.1 ± 2.17
Sucrose (mg/g flour)	73.3 ± 0.78
Glucose (mg/g flour)	79.6 ± 1.13
Fructose (mg/g flour)	195 ± 0.22
Total polyphenols (mg GAE/g flour)	17.02 ± 3.18

Values are presented as means ± DE (*n*=3). GAE, gallic acid.

**Table 2:** Effects of yacon supplement on serum lipid profile of HFD-fed rats.

	SD	HFD	HFD Y340	HFD Y680
Triglyceride (mg/dl)	64.1 ± 5.0	195.8 ± 12.1^a^	98.5 ± 2.4^b^	83.0 ± 4.1^b^
Total cholesterol (mg/dl)	70.2 ± 14.1	86.2 ± 26.0	80.2 ± 32.0	79.1 ± 18.0
HDLc (mg/dl)	48.0 ± 5.1	32.4 ± 10.3^a^	40.6 ± 14.2	50.3 ± 7.2^b^
LDLc (mg/dl)	13.6 ± 8.2	44.2 ± 9.5^a^	27.2 ± 8.1	19.3 ± 6.5^b^
TG/HDLc index	1.33 ± 0.11	6.09 ± 1.04^a^	2.47 ± 0.07^b^	1.67 ± 0.16^b,c^
Free fatty acid (mmol/l)	0.76 ± 0.11	1.10 ± 0.13^a^	0.96 ± 0.05^b^	0.84 ± 0.07^b,c^

Values are means ± standard deviation (*n*=6 rats/group). ^a^ *p* < 0.05 compared to the SD group. ^b^ *p* < 0.05 compared to the HFD group.

**Table 3:** Effects of yacon supplement on organs weights of HFD-fed rats.

	SD	HFD	HFD Y340	HFD Y680
Total fat pad (% b. w.)	3.40 ± 0.69	6.44 ± 0.67^a^	5.54 ± 0.53	3.61 ± 0.95^b^
Epididymal fat pad (% b. w.)	1.47 ± 0.13	2.18 ± 0.20^a^	1.94 ± 0.26	1.42 ± 0.22^b^
Perirenal fat pad (% b. w.)	1.06 ± 0.26	2.55 ± 0.37^a^	2.15 ± 0.23	1.47 ± 0.31^b^
Visceral fat pad (% b. w.)	0.86 ± 0.23	1.71 ± 0.18^a^	1.33 ± 0.12^b^	0.72 ± 0.22^b^
Muscle (soleus) (% b. w.)	1.35 ± 0.07	0.98 ± 0.20^a^	1.20 ± 0.19	1.18 ± 0.26
Liver (% b. w.)	2.66 ± 0.13	3.10 ± 0.18^a^	2.51 ± 0.11^b^	2.47 ± 0.12^b^
Spleen (% b. w.)	0.13 ± 0.01	0.12 ± 0.02	0.13 ± 0.01	0.13 ± 0.03
Pancreas (% b. w.)	0.43 ± 0.11	0.43 ± 0.12	0.49 ± 0.02	0.58 ± 0.06
Cecum (% b. w.)	0.23 ± 0.02	0.16 ± 0.02^a^	0.20 ± 0.03	0.28 ± 0.04^b,c^

Values are means ± standard deviation (*n*=6 rats/group). ^a^ *p* < 0.05 compared to the SD group. ^b^ *p* < 0.05 compared to the HFD group; ^c^ *p* < 0.05 compared to the HFD Y340 group. Fat pad weights are expressed as the sum of visceral, retroperitoneal, and epididymal weights. b. w., body weight.

## Supplementary Materials

Supplementary Materials Figure S1 provides evidence that yacon supplementation modifies lipid absorption at the intestinal level through an oral triglyceride loading test. Table S1 shows the effect of yacon on morphometric parameters after HFD rats turned to SD feeding for 8 weeks. Figure S2 provides evidence that yacon improves nutritional parameters after HFD rats turned to a SD feeding.
