# Supplementary material for: Smallanthus sonchifolius (Yacon) Flour Improves Visceral Adiposity and Metabolic Parameters in High-Fat-Diet-Fed Rats
Source: J Obes. 2018 Oct 28;2018:5341384. doi: 10.1155/2018/5341384 (PMC6230400; doi:10.1155/2018/5341384)
Supplement: Supplementary Materials — Figure S1 provides evidence that yacon supplementation modifies lipid absorption at the intestinal level through an oral triglyceride loading test. Table S1 shows the effect of yacon on morphometric parameters after HFD rats turned to SD feeding for 8 weeks. Figure S2 provides evidence that yacon improves nutritional parameters after HFD rats turned to a SD feeding. [file 5341384.f1.pdf]

Supplementary material

Table S1. Effects of yacon on morphometric parameters of HFD–fed rats

	SD	HFD	HFD Y340	HFD Y680
Final body weight (g)	429.8 ± 21.0	615.4 ± 28.8[a]	572.77±10.12[a,b]	520.28±23.52[a,b,c]
BMI (%)	0.71 ±0.09	0.95 ± 0.02[a]	0.98±0.05[a]	0.81±0.03[a,b]
Lee index (g/cm)	0.31 ± 0.01	0.34 ± 0.01[a]	0.35±0.01[a]	0.32±0.01[b]
TC (cm)	17.50 ± 0.50	21.88 ± 1.70[a]	18.50 ± 1.06[b]	17.25±0.57[a,b,c]
AC (cm)	17.33 ± 1.04	22.20 ± 1.24[a]	19.33 ± 0.71[a,b]	17.75±0.57[a,b,c]
AC/TC	1.01 ± 0.02	1.09 ± 0.05[a]	1.04 ± 0.03	0.98 ± 0.01[b]

Values are means ± standard deviation (n = 6 rats/group). [a] $p < 0.05$ compared to the SD. [b] $p < 0.05$ compared to the HFD. TC, thoracic circumference; AC, abdominal circumference

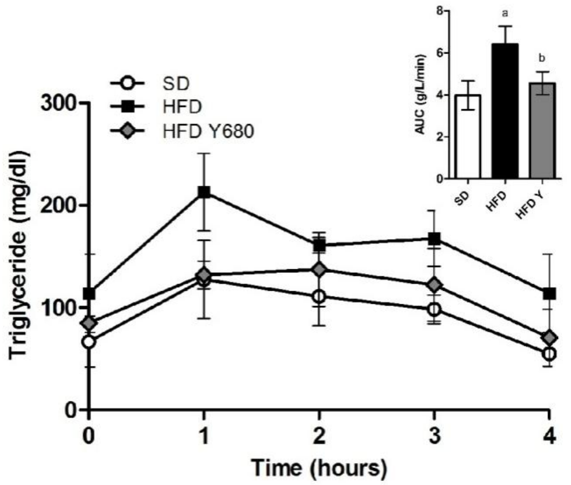

Figure S1. **Effects of yacon flour on serum triglyceride after oral fat-loading in HFD-fed rats.** Time course of triglycerides after a single yacon-tablet (680 mg FOS/kg body weight) followed of normal standard chow administration at 0,1,2,3 and 4 h (Insert: area under the curve of serum triglycerides following 2.5 ml/ kg b.w. of corn oil administration). Data are expressed as the mean ± standard deviation (n = 6 / group). [a]$p < 0.05$ vs. SD, [b]$p < 0.05$ vs. HFD. SD: Standard-diet fed rats; HFD: High-fat diet- fed rats; HFD Y680: High-fat diet- fed rats supplemented with yacon flour (680 mg FOS/kg body weight).

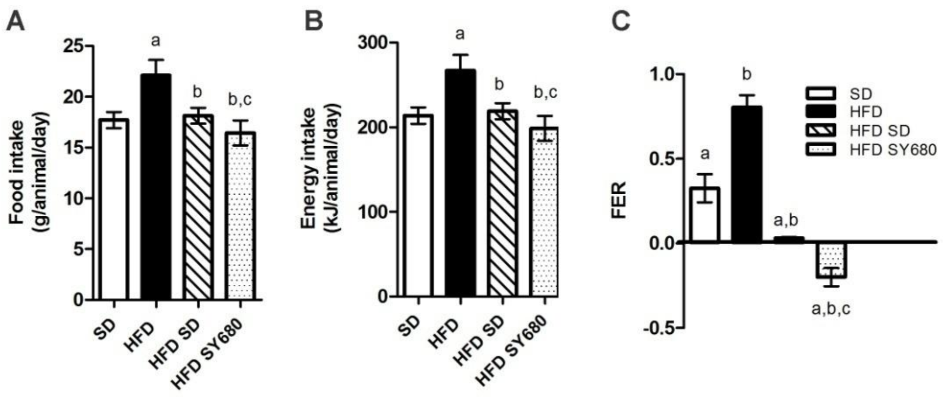

Figure S2. **Effects of yacon supplement and the reversion to a SD diet on HFD-induced metabolic disease.** A: Food intake. B: Energy intake. C: Feed efficiency ratio (FER). Data are mean ± standard deviation (n= 6 / group). [a]p < 0.05 vs. SD, [b]p < 0.05 vs. HFD, [c]p < 0.05 vs. HFD SD. SD: Standard-diet fed rats; HFD: High-fat diet- fed rats; HFD SD: High-fat diet- fed rats reversed to a SD diet; HFD SY680: High-fat diet- fed rats reversed to a SD diet and supplemented with yacon flour (680 mg FOS/kg b. w.).
